# Comparison of pre-analytical conditions for quantification of serotonin in platelet-poor plasma

**DOI:** 10.1016/j.plabm.2019.e00136

**Published:** 2019-09-19

**Authors:** Hilde L. Von Volkmann, Ingeborg Brønstad, Torunn Fiskerstrand, Oddrun Anita Gudbrandsen

**Affiliations:** a National Centre for Ultrasound in Gastroenterology, Department of Medicine, Haukeland University Hospital, Bergen, Norway; b Department of Clinical Medicine, University of Bergen, Bergen, Norway; c Center for Medical Genetics and Molecular Medicine, Haukeland University Hospital, Bergen, Norway; d Department of Clinical Science, University of Bergen, Bergen, Norway

**Keywords:** Serotonin, 5-HT, 5-Hydroxytryptamine, Plasma, Platelet-poor plasma, Pre-analytical procedure

## Abstract

**Background:**

Reported concentrations of serotonin in platelet-poor plasma (PPP) in healthy subjects vary widely due to different pre-analytical procedures.

**Aim:**

To examine how different pre-analytical conditions affect the measured concentration of serotonin in PPP.

**Method:**

Six pre-analytical protocols were compared for preparation of PPP from EDTA whole blood for quantification of serotonin from nine healthy individuals. Three combinations of centrifugation with a mild centrifugation of gel-free EDTA tubes followed by a stronger centrifugation were compared to single-stage centrifugation of EDTA tubes with separator gel and heat shock treatment of blood prior to centrifugation. All samples were analysed using the same enzyme linked immunosorbent assay (ELISA) method.

**Results:**

Findings show that two consecutive centrifugations; first a mild centrifugation at 100 or 200×*g* followed by centrifugation at 4500 or 14500×*g* resulted in the lowest serotonin concentration in PPP.

**Conclusion:**

Two successive centrifugations to produce PPP for serotonin analysis; first a mild centrifugation to avoid mechanical stress on the platelets, and next a stronger centrifugation to remove platelets, is superior to the use of gel tubes and heat shock treatment.

## 1 Introduction

Serotonin (5-hydroxytryptamine) is an important neurotransmitter and neuromodulator which affects many neurophysiological processes, mood and cognition, regulates motility and sensibility in the bowel, and has an essential role in regulating blood pressure [1]. Merely 1–2% of the essential amino acid L-tryptophan from dietary intake is metabolized to serotonin in humans, whereas most of the ingested L-tryptophan is metabolized through the kynurenine pathway [2]. Serotonin in blood is primarily originating from biosynthesis in the gastrointestinal tract, and after secretion serotonin is rapidly taken up and is transported in platelets, with free circulating serotonin representing only a minor fraction of the total serotonin amount in blood [3].

Since only free plasma serotonin can bind to serotonin receptors and exert biological activity [4], it is important to have a reliable and simple pre-analytical procedure to prepare platelet-poor plasma (PPP) for quantification of non-platelet bound serotonin. After collection of blood, platelets are exposed to both mechanical and thermal stress; several aspects such as the cannula diameter, blood flow, anti-coagulant, pipetting, change in temperature and conditions during centrifugation (temperature, force and time) may affect the release of serotonin from the blood platelets [5,6]. As a consequence of this, reported concentrations of serotonin in PPP may vary widely due to differences in pre-analytical procedures [4]. The aim of this investigation was to examine how different pre-analytical conditions may affect the measured concentration of serotonin in PPP from healthy volunteers, and to identify the pre-analytical protocol resulting in the lowest PPP serotonin concentration.

## 2 Methods

### 2.1 Participants, study setting and ethics

Blood samples were collected from nine healthy adults recruited at Haukeland University Hospital, Bergen, Norway in September 2015. Examinations were conducted at Haukeland University Hospital. The study was conducted according to the guidelines laid down in the Declaration of Helsinki and all procedures were approved by the Regional Committee for Medical and Health Research Ethics of Western Norway (REC-number: 2014/2222). Written informed consent was obtained from all subjects. All data were analysed anonymously.

### 2.2 Protocol for study visits

The participants attended one study visit. Examinations were conducted in the morning after an overnight fast. The subjects were instructed not to eat or drink anything except water after 22.00 the previous day, to avoid intake of serotonin-rich food such as bananas, ice-cream, nuts, chocolate, tomato and kiwi for 72 h before the visit, to avoid drinking coffee or alcohol and smoking cigarettes for 24 h prior to the visit. Blood was drawn from an antecubital vein and was collected in 3 ml VACUETTE® K2EDTA tubes and 5 ml VACUETTE® K2EDTA gel tubes (Greiner Bio-one) for isolation of plasma, all tubes had a final EDTA concentration of 4.429 mmol/L.

### 2.3 Preparation of platelet-poor plasma (PPP)

PPP was prepared separately from blood samples from each of nine participant using six different pre-analytical procedures as shown in Table 1. Blood was collected in tubes in the order A to F for each sampling. All blood samples were centrifuged within 5 min after collection, and PPP was stored at −80 °C until analysed.

**Table 1:** Overview of the preparation methods of platelet-poor plasma for serotonin analyses.

Pre-analytical method	Sampling tube	First centrifugation (EDTA blood)	Second centrifugation (supernatant)	References
A	3 ml VACUETTE® K2EDTA tube	200×*g* 10 min room temperature	4500×*g* 10 min 4 °C	Recommended by IBL [7]
B	3 ml VACUETTE® K2EDTA tube	150×*g* 20 min room temperature	550×*g* 15 min room temperature	Yubero-Lahoz [8]
C	3 ml VACUETTE® K2EDTA tube	100×*g* 15 min 4 °C	14.500×*g* 6 min 4 °C	Frost [9]
D	3 ml VACUETTE® K2EDTA tube	Heat shock: 42 °C for 10min, then 1800×*g* 15 min 4 °C	none	Maurer-Spurej [11]
E	5 ml VACUETTE® K2EDTA gel tube	1800×*g* 15 min 4 °C	none	
F	5 ml VACUETTE® K2EDTA gel tube	1800×*g* 15 min 22 °C	none	

### 2.4 Analyses of serotonin in PPP

Serotonin was quantified using competitive ELISA (#RE59121, IBL International GmbH, Hamburg, Germany). Acylated samples were incubated overnight with serotonin biotin and serotonin antiserum (rabbit) on a microtiter plate coated with anti-rabbit antiserum. Bound biotinylated serotonin was determined by use of streptavidin alkaline phosphatase (marker) and *p*-nitrophenyl phospahte (substrate). Samples were analysed in duplicate or triplicate. The inter assay coefficient of variation, which was tested using a quality control solution (13.2 ng/mL, provided with the kit) in triplicate from 3 plates, was 7.4%, and the mean coefficient of variation for each PPP sample within each assay was <5%, which were within the ranges indicated by the manufacturer [7].

### 2.5 Statistical analyses

Statistical analyses were conducted using SPSS Statistics 25 (SPSS, Inc., IBM Company). Normality of data was tested by Shapiro Wilks test. Measured serotonin concentrations were compared using one-way ANOVA followed by Tukey post-hoc test. Data are expressed as mean with standard deviation (SD). P < 0.05 was considered statistically significant.

## 3 Results

### 3.1 Comparison of different pre-analytical conditions on PPP serotonin concentrations

Table 2 shows the measured serotonin concentrations in PPP with different pre-analytical conditions. Preparation methods were compared using one-way ANOVA (*p* 1.2 × 10^−12^) and the Tukey post-hoc test demonstrated that serotonin concentrations in PPP prepared by method E was significantly different from PPP prepared by all other methods (*p* < 1 × 10^−7^ for all individual comparisons including method E, data not presented). To achieve a better discrimination between results from the other pre-analytical methods, results from method E was omitted and the ANOVA analysis was repeated (*p* ANOVA 1.4 × 10^−4^). This showed that serotonin concentrations were significantly lower in PPP prepared by methods A, B and C when compared to those prepared by methods D and F, with no significant differences between measured serotonin concentrations between methods A, B and C, and no significant differences between methods D and F.

**Table 2:** Measured serotonin concentrations in platelet-poor plasma from healthy donors (N = 9).

Pre-analytical method	Measured Serotonin Concentration (ng/mL)^1^	
A	0.9 ± 0.5^a^	
B	2.5 ± 2.3^a^	
C	1.0 ± 0.5^a^	
D	9.8 ± 11.1^b^	
E	41.4 ± 18.6	
F	10.7 ± 1.6^b^	

Serotonin concentration is presented as mean ± standard deviation. Serotonin concentrations in PPP prepared by method E was discarded from statistical analysis since results deviated strongly from results obtained with the other pre-analytical methods.

^1^Methods A, B, C, D and F are compared using one-way ANOVA (*p* = 1.4 × 10^−4^) with Tukey post-hoc test; means in a column with different letters are significantly different (p < 0.05).

## 4 Discussion

This is the first study to compare different centrifugation conditions on measured serotonin concentrations in PPP from healthy volunteers, without varying blood sampling techniques and type of anticoagulant added to identify the most suitable pre-analytical conditions for PPP serotonin quantification by ELISA. Three previously reported methods using double-stage centrifugation [[7], [8], [9]] and three alternative singel-stage centrifugation methods using separation gel or heat shock treatment for preparing PPP were compared. This work demonstrates that a combination of a milder first centrifugation of gel-free EDTA-tubes to separate blood cells (100 or 200×*g*) followed by a stronger centrifugation of the supernatant (4500 or 14500×*g*) resulted in the lowest measured serotonin concentration in PPP.

It is well known that the liberation of serotonin from platelets to plasma is affected by both thermal and mechanical conditions [5,6], and as a consequence reported serotonin concentrations in PPP vary widely in the literature [4]. Comparisons of different pre-analytical and analytical factors on the measured PPP serotonin concentrations have been published [4,8]. In the present study we compare different pre-analytical protocols for PPP from healthy adults before analyses using ELISA, thus making it easier to identify the best pre-analytical protocol for quantification of serotonin in PPP. In the largest overview of published concentrations of serotonin in PPP from healthy subjects published to date, Brand and Anderson [4] demonstrate a huge variation in reported serotonin values from 101 studies, ranging from 0.1 to 31.6 ng/mL, and the authors suggest an upper limit of PPP serotonin of 1 ng/ml. However, since the analytical method in use strongly impacted the resulting serotonin concentration, e.g., radioenzymatic assay and mass spectrometry typically produce lower results compared to ELISA, this upper limit should be interpreted with great care. In the present study we compared different pre-analytical methods described in publications that presents low serotonin concentrations in PPP from healthy individuals; Yubero-Lahoz et al. [8] reporting a mean serotonin PPP concentration of 0.62 ± 0.12 ng/mL (N = 15), Lederer et al. [10], who prepared PPP as advised by IBL [7], reported a mean plasma serotonin concentration of 0.11 ng/mL (N = 6) and Frost et al. [9] reported mean serotonin PPP concentration of 3.51 **±** 0.49 ng/mL (N = 18). We do however suspect that there may be a calculation error in the paper by Lederer et al. [10], since Lederer et al. give the value as 1.3 nmol/L when discussing the 97th percentile value given in the IBL kit literature, when it is actually stated as 7.5 ng/mL by IBL (equal to 42.5 nmol/L, using the correct conversion factor of 5.67 to go from ng/mL to nmol/L). Thus, it appears that when converting from ng/mL to nmol/L, Lederer et al. divided their ng/mL results by 5.67 instead of multiplying by 5.67, and thus, the reported value of Lederer of 0.63 nmol/L (~0.11 ng/mL) was probably actually 20 nmol/L (~3.5 ng/mL).

The protocol with heat shocking of the platelets were inspired by the paper by Maurer-Spurej et al. [11], who demonstrated that moderate heat shock conditions (42 °C for 10 min) caused no signs of platelet activation, whereas room temperature causes marked activation of platelets and affected platelet serotonin content.

Plasma serotonin concentration has been shown to increase by >300% after 40 min on ice compared to immediate centrifugation [12]. This motivated us to test EDTA-tubes with gel to achieve rapid separation of blood cells and plasma with only one centrifugation (1800×*g* for 15min, with temperatures 4 °C or 22 °C), as these gel tubes are commonly used in the clinic and would be convenient to implement in the daily routine.

Our results show that two consecutive centrifugations resulted in the lowest measured serotonin concentrations in PPP (methods A, B and C), probably because these protocols include is a milder method for separation of blood cells in the first centrifugation step (100-200×*g*) that causes less stress on the blood platelets and thus less serotonin liberation from the platelets. Of these three methods tested, methods A and C seem to be the superior methods, probably because the second centrifugation (4500 and 14500×*g*, respectively) removed platelets to a larger degree compared to method B (550×*g*), Thus, the lowest measured serotonin mean concentration were 0.9 (SD 0.5) and 1.0 (SD 0.5) ng/mL, for method A and C, respectively. The use of heat shock (method D) did not prevent serotonin liberation from the platelets, and measured serotonin values were higher when compared to methods A, B and C.

The use of EDTA tubes with separator gel resulted in very high serotonin concentrations when gel tubes were centrifuged at 4 °C (method E), with large variations between the samples. Also, when gel tubes were centrifuged at 22 °C (method F), mean serotonin concentration was higher compared to methods A, B and C, but concentration was lower compared to method E, however with small variations between the samples. These findings suggest that either the use of gel tubes somehow activated the platelets, or that the gel was not sufficiently effective in separating blood platelets from plasma, especially when centrifuged at 4 °C.

In conclusion, two consecutive centrifugations of EDTA-blood; first a mild centrifugation at 100 or 200×*g* followed by a stronger centrifugation at 4500 or 14500×*g* at 4 °C, resulted in the lowest PPP serotonin concentrations, regardless of whether first centrifugation temperature was 4 °C or 22 °C, and are superior to the use of gel tubes and heat shock treatment. Our interpretation is that the first centrifugation induced little mechanical stress on the blood platelets and the second centrifugation was sufficiently strong to prevent contamination of platelets in the PPP fraction.

## Declarations of interest

None.

## Appendix A Supplementary data

Dataprofile Dataprofile
